# Supplementary material for: Long lived surface plasmons on the interface of a metal and a photonic time-crystal
Source: Nanophotonics. 2025 Mar 11;14(23):4063–8. doi: 10.1515/nanoph-2024-0735 (PMC12617727; doi:10.1515/nanoph-2024-0735)
Supplement: Supplementary file 1 — Supplementary Material Details [file j_nanoph-2024-0735_suppl_001.docx]

**Supplementary Information for**

**Surface plasmons on the interface of a metal and a photonic time-crystal**

Lior Bar-Hillel^1+^, Yonatan Plotnik^2^, Ohad Segal^1^ and Mordechai Segev^1,2^

1. *Department of Electrical and Computer Engineering, Technion, Haifa 32000, Israel*
2. *Physics Department, Technion, Haifa 32000, Israel*

**Section A: Derivation of the band structure of a PTC**

In this section, we derive the EM modes within a PTC and its band structure. Although this was done in the past in many papers [2,3, 6-8], we nonetheless present it here, for completeness. We start by studying the EM mode within a PTC. We assume a PTC with relative permittivity $\varepsilon\left( t+\frac{2\pi}{\Omega} \right)=\varepsilon\left( t \right)$ which can be expanded as a Fourier series $\varepsilon\left( t \right)=\sum_{n} \varepsilon_{n}e^{-in\Omega t}$. Within the PTC, Maxwell’s curl equations take the form

|  | $\vec{\nabla}\times\vec{E}=-\mu_{0}\partial_{t}\vec{H}, \vec{\nabla}\times\vec{H}=\partial_{t}\left( \varepsilon_{o}\varepsilon\left( t \right)\vec{D} \right),$ | (A1) |
| --- | --- | --- |

where we assumed that the responses of the material is instantaneous. We apply a curl operator on Ampere’s law and use the vector identity $\vec{\nabla}\times\left( \vec{\nabla}\times\vec{V} \right)=\vec{\nabla}\left( \vec{\nabla}\cdot\vec{V} \right)-\vec{\nabla}^{2}\vec{V}.$ We substitute the Faraday law and the Gauss law for the magnetic flux, $\vec{\nabla}\cdot\vec{B}=\mu_{0}\vec{\nabla}\cdot\vec{H}=0,$ and find the wave equation for the magnetic field

|  | $\vec{\nabla}^{2}\vec{H}=\frac{1}{c^{2}}\partial_{t}\left( \varepsilon\left( t \right)\partial_{t}\vec{H} \right).$ | (A2) |
| --- | --- | --- |

According to the Bloch-Floquet theorem, the magnetic field must be of the form $H_{y}=\sum_{n} h_{n}\left( \omega\right)e^{i\left( \vec{k}\cdot\vec{r}-\omega_{n}t \right)}$, where we denote $\omega_{n}=\omega+n\Omega$. Substituting the magnetic field into Eq. A2 results in an eigenvalue problem

|  | $\sum_{n} \omega_{n}\omega_{l}\varepsilon_{l-n}h_{n}\left( \omega\right)=c^{2}k^{2}h_{l}\left( \omega\right).$ | (A3) |
| --- | --- | --- |

Equation A3 can be rearranged in matrix form $D\left( \omega\right)\vec{h}=c^{2}k_{PTC}^{2}\left( \omega\right)\vec{h}$, where $\left[ D \right]_{n,m}=\varepsilon_{m-n}\omega_{n}\omega_{m}$. We find that the eigenvalues and eigenvectors of $D$ yield the band structure $k_{b,PTC}\left( \omega\right)$ and the EM mode $h_{bn}\left( \omega\right),$ respectively. $D$ has infinite number of rows and columns, thus, when preforming numerical calculations, we truncate $D$ to be $N\times N$ matrix, resulting in $N$ bands which are the $N$ eigenvalues of the matrix. For the next section, it is important to note that this derivation holds for a general wave-vector even if it has complex components.

**Section B: Derivation of dispersion curve and eigenfunctions of SPPs**

We consider a system as presented in Fig. 1, with a PTC on one side and a plasmonic metal on the other side. We guess a solution for the magnetic field that satisfies the wave equation in each side of the interface. The most general magnetic field with frequency $\omega$ within the PTC, must be a sum over modes from different bands with the same frequency. Thus, a TM mode that accumulate phase along the interface while decaying in the $\pm z$ direction must take the form

|  | $H_{y}\left( \vec{r},t \right)=\sum_{b=1}^{\infty} A_{b}\sum_{n=-\infty}^{\infty} h_{bn}\left( \omega\right)e^{i\left( k_{spp}x-\omega_{n}t \right)}\left\{ \begin{aligned} e^{-\kappa_{b,PTC}z}, z>0 \\ e^{\bar{\kappa_{n,m}}z}, z<0 \end{aligned} \right..$ | (B1) |
| --- | --- | --- |

Where the complex conjugate of $\kappa_{n,m}$ is necessary for a correct direction of propagation for $k_{spp}<k_{m}\left( \omega_{n} \right)$. The corresponding tangential electric field is derived from the equation $\vec{\nabla}\times\vec{E}=-\mu_{0}\frac{\partial\vec{H}}{\partial t}$ and is given by

|  | $E_{x}=\sum_{b=1}^{\infty} \sum_{n=-\infty}^{\infty} A_{b}h_{bn}\left( \omega\right)\mu_{0}\omega_{n}e^{i\left( k_{spp}x-\omega_{n}t \right)}\left\{ \begin{aligned} \frac{i\kappa_{b,PTC}}{k_{b,PTC}^{2}\left( \omega_{n} \right)}e^{-\kappa_{b,PTC}z}, z>0 \\ \frac{-i\bar{\kappa_{n,,m}}}{k_{m}^{2}\left( \omega_{n} \right)}e^{\bar{\kappa_{n,m}}z}, z<0 \end{aligned} \right..$ | (B2) |
| --- | --- | --- |

For completeness, the z component of the electric field is given by

|  | $E_{z}=\sum_{b=1}^{\infty} \sum_{n=-\infty}^{\infty} -k_{spp}A_{b}h_{bn}\left( \omega\right)\mu_{0}\omega_{n}e^{i\left( k_{spp}x-\omega_{n}t \right)}\left\{ \begin{aligned} \frac{1}{k_{b,PTC}^{2}\left( \omega_{n} \right)}e^{-\kappa_{b,PTC}z}, z>0 \\ \frac{1}{k_{m}^{2}\left( \omega_{n} \right)}e^{\bar{\kappa_{n,m}}z}, z<0 \end{aligned} \right..$ | (B3) |
| --- | --- | --- |

Next, we impose boundary conditions on the electric field along the interface. The electric field must be continuous along the interface, thus

|  | $\sum_{n=-\infty}^{\infty} \omega_{n}e^{i\left( k_{spp}x-\omega_{n}t \right)}\sum_{b=1}^{\infty} \left( \frac{\bar{\kappa_{n,m}}}{k_{m}^{2}\left( \omega_{n} \right)}+\frac{\kappa_{b,PTC}}{k_{b,PTC}^{2}\left( \omega_{n} \right)} \right)h_{bn}\left( \omega\right)A_{b}=0.$ | (B4) |
| --- | --- | --- |

Equation B4 must hold for every point x along the interface and for all time t, which leads to Eq. 2 in the main text. For $\varepsilon_{r}=const$ the eigenmodes are $h_{bn}=\delta_{n,0}$ leading to the known dispersion of SPP folded into the region $-\frac{\Omega}{2}\leq\omega\leq\frac{\Omega}{2}$.

**Section C: Simulation results for a non-ideal metal**

For the completeness of the study presented in the main text, we simulate the propagation of SPPs at an interface of a PTC and a metal with energy dissipation. We model the metal using the Drude model with decay time $\tau$. To emphasize the importance of our findings, we take $\tau=2fs$ which leads to much faster energy dissipation than the dissipation in metals such as silver and gold. We simulate a pulse, propagating along the interface. At time $t=50$ the PTC is turned on, where the permittivity of the PTC is a square wave between $\varepsilon_{l}=1$ and $\varepsilon_{m}$ (as in Fig. 1(a)). The frequency of the pulse is chosen to be half of the modulation frequency. The results for several modulation amplitudes and periods are presented in Fig. 5. The energy of the SPP pulse as a function of time for different parameters is presented in Fig. 5(c) and (d).


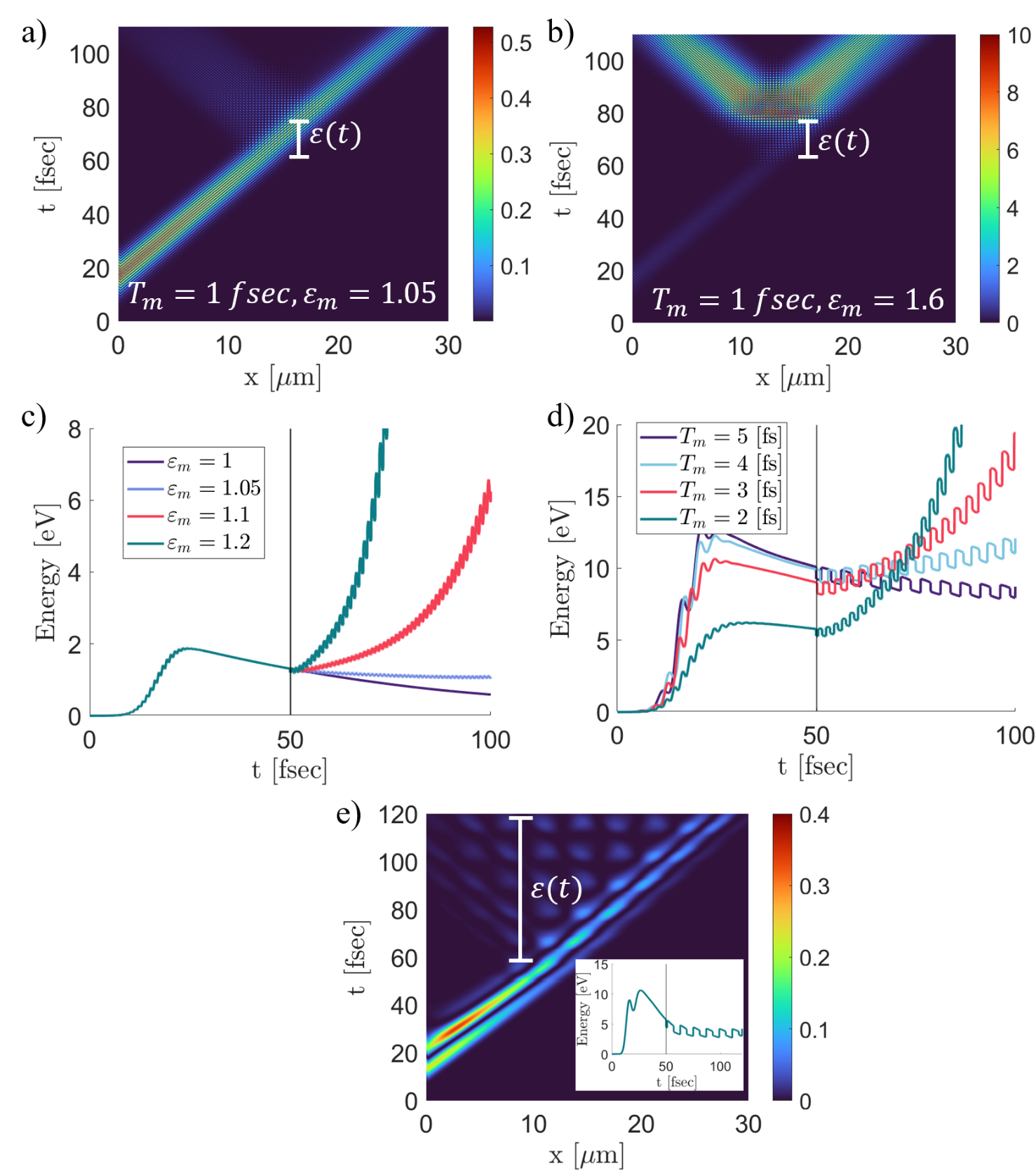


Fig. 5: **FDTD simulations of SPPs at the interface between a PTC and a realistic metal for different modulation parameters.** (a), (b) The magnetic field [a.u.] along the interface as a function of time, $H_{y}\left( x,z=0,t \right)$, for two values of$\varepsilon_{m}$ and $\varepsilon_{l}=1$. The PTC is on at time interval 60-80 fsec. The period of the PTC is $T_{m}=1fsec$ and its peak permittivity is 1.05 and 1.6 respectively. (c) EM energy of the pulse as a function of time for several modulation amplitudes. The PTC turns on at $t=50 fsec$, which is marked by a vertical black line, and its period is $T=1 fsec$. (d) EM energy of the pulse as a function of time for several modulation periods. The PTC turns on at $t=50 fsec$, which is marked by a vertical black line, and its peak permittivity is $\varepsilon_{m}=1.2$. In each simulation, the main frequency of the pulse is chosen to be half the modulation frequency. (e) A similar simulation to those in (a) and (b) with energy dissipation rate of $\tau=10fsec$ in the metal and a value of $\frac{\varepsilon_{m}}{\varepsilon_{l}}=0.8$ which matches the permittivity variation measured in [12]. The period of the modulation is taken to be $T=12 fsec$ which balances the plasmonic losses.

We see that the amplification of the PTC can overcome plasmonic losses if the modulation is fast enough or strong enough. Specifically, the blue curve in Fig. 5(c) and purple curve in Fig. 5(d) indicates modulation parameters for which the amplification balances the plasmonic losses. Moreover, Fig. 5(e) shows a case for which the amplification balances the plasmonic losses for a realistic metal with $\tau=10fsec$. We choose the period of the modulation in Fig. 5(e) according to the following reasoning: For a modulation of the form $\varepsilon\left( t \right)=\varepsilon_{1}+\varepsilon_{2}\left( 1+\cos\Omega t \right)$, the condition for overcoming the losses which was estimated in the main text is $\frac{5\varepsilon_{1}}{\varepsilon_{2}}<\tau\Omega$. For a realistic metal, which has energy dissipation time of $\sim10 fsec$ and a ratio of maximum and minimum permittivity that matches to the measured on in [12] which is $\frac{\varepsilon_{m}}{\varepsilon_{l}}\sim0.8$, the period of the PTC that result in amplification that balances the plasmonic losses is $\sim12 fsec$. We find that this period enables the amplification to overcome the plasmonic losses, Fig. 5(e). To summarize, by strengthen the modulation of the PTC or to shorten its period, we can take advantage of the amplification that is inherited in PTCs, and overcome plasmonic loss of SPPs.
